# Supplementary material for: An improved fluorescent tag and its nanobodies for membrane protein expression, stability assay, and purification
Source: Commun Biol. 2020 Dec 10;3:753. doi: 10.1038/s42003-020-01478-z (PMC7729955; doi:10.1038/s42003-020-01478-z)
Supplement: Supplementary file 1 — Supplementary Information [file 42003_2020_1478_MOESM1_ESM.pdf]

SUPPLEMENTARY INFORMATION

Supplementary figures 1-11
Supplementary References 1-3.

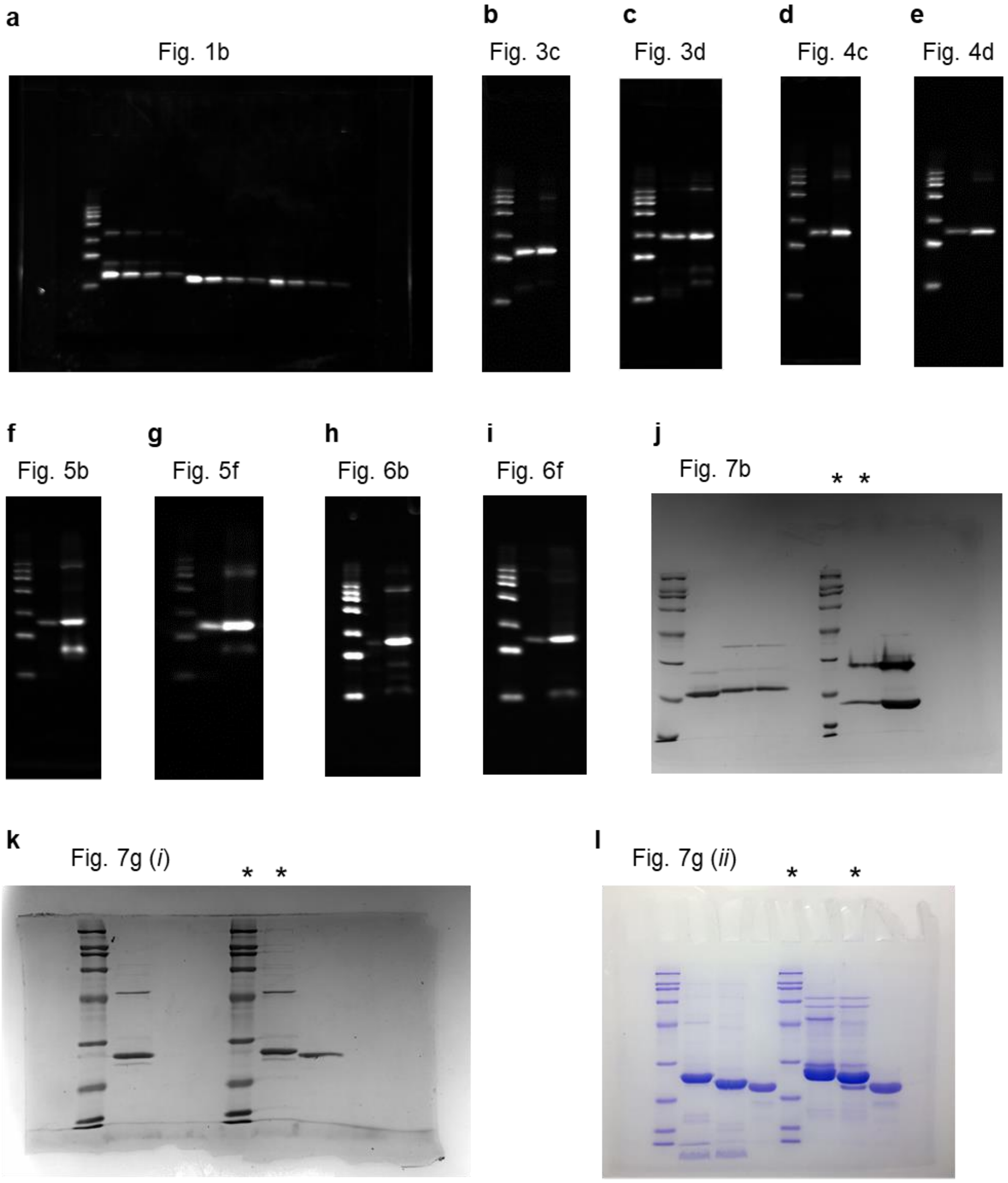

Supplementary Fig. 1. Uncropped images of panels in the main figure. (**a-i**) In-gel fluorescence images without inverting the colors. The corresponding main figure panel is labeled on top of each image. (**j-l**) Full image of the main figure panels as indicated. Asterisks denote lanes appeared in the main figure.

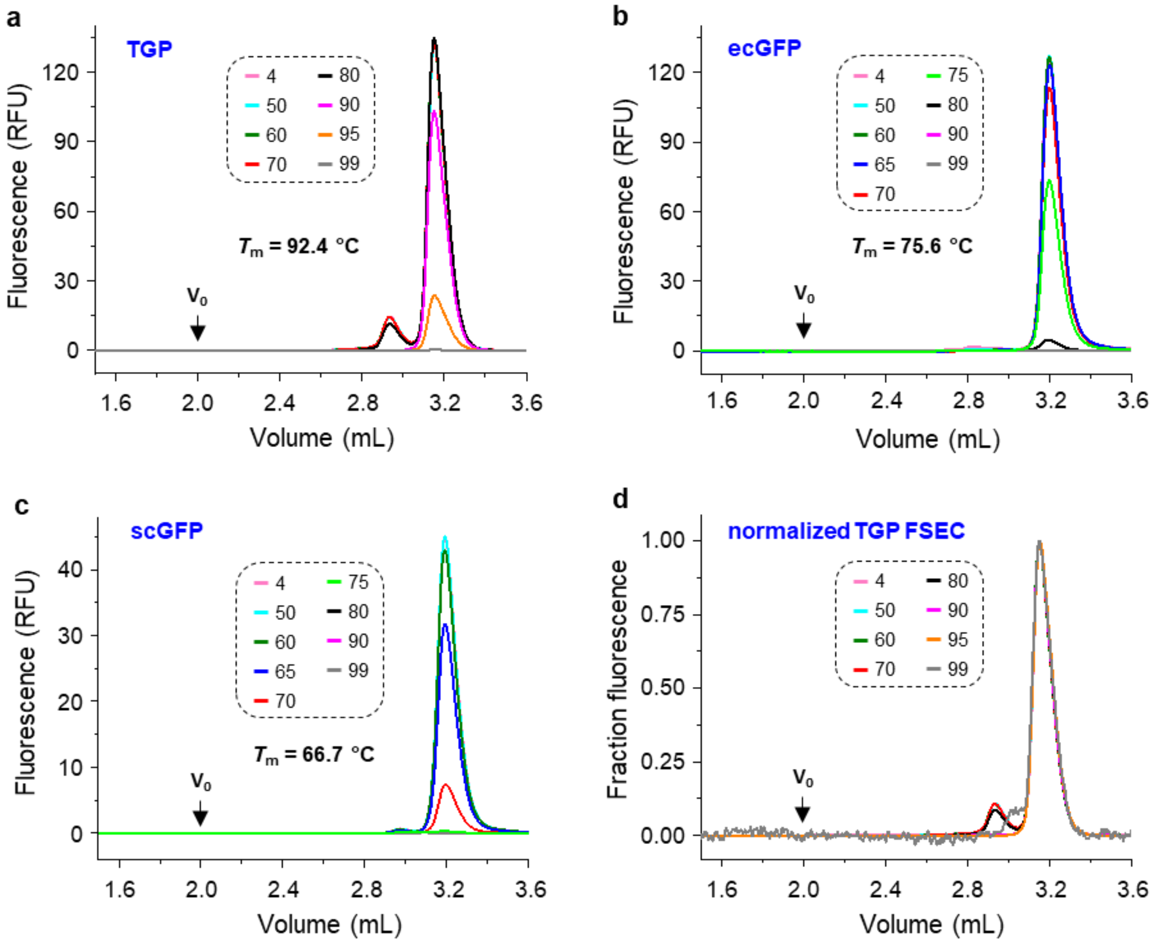

Supplementary Fig. 2. Original FSEC profiles for the FSEC-TS assay of fluorecent proteins. (**a-c**) Unnormalized FSEC traces of the indicated fluorecent proteins heated at temperatures shown in the dashed box. (**d**) Superimposable normalized FSEC traces of TGP heated under different temperatures. The left part (baseline) of the chromatography is trimmed for all FSEC figures. The same color scheme for temperatures (when applicable) was used for different fluorecent proteins, as indicated in the dashed boxes. Abbreviations: FSEC, fluorescence-detection size exclusion chromatography; FSEC-TS, FSEC-based thermostability assay; GFP, green fluorescent protein; TGP, thermostable GFP; V_o, void volume; V_t, total volume.

Related to Fig. 1e.

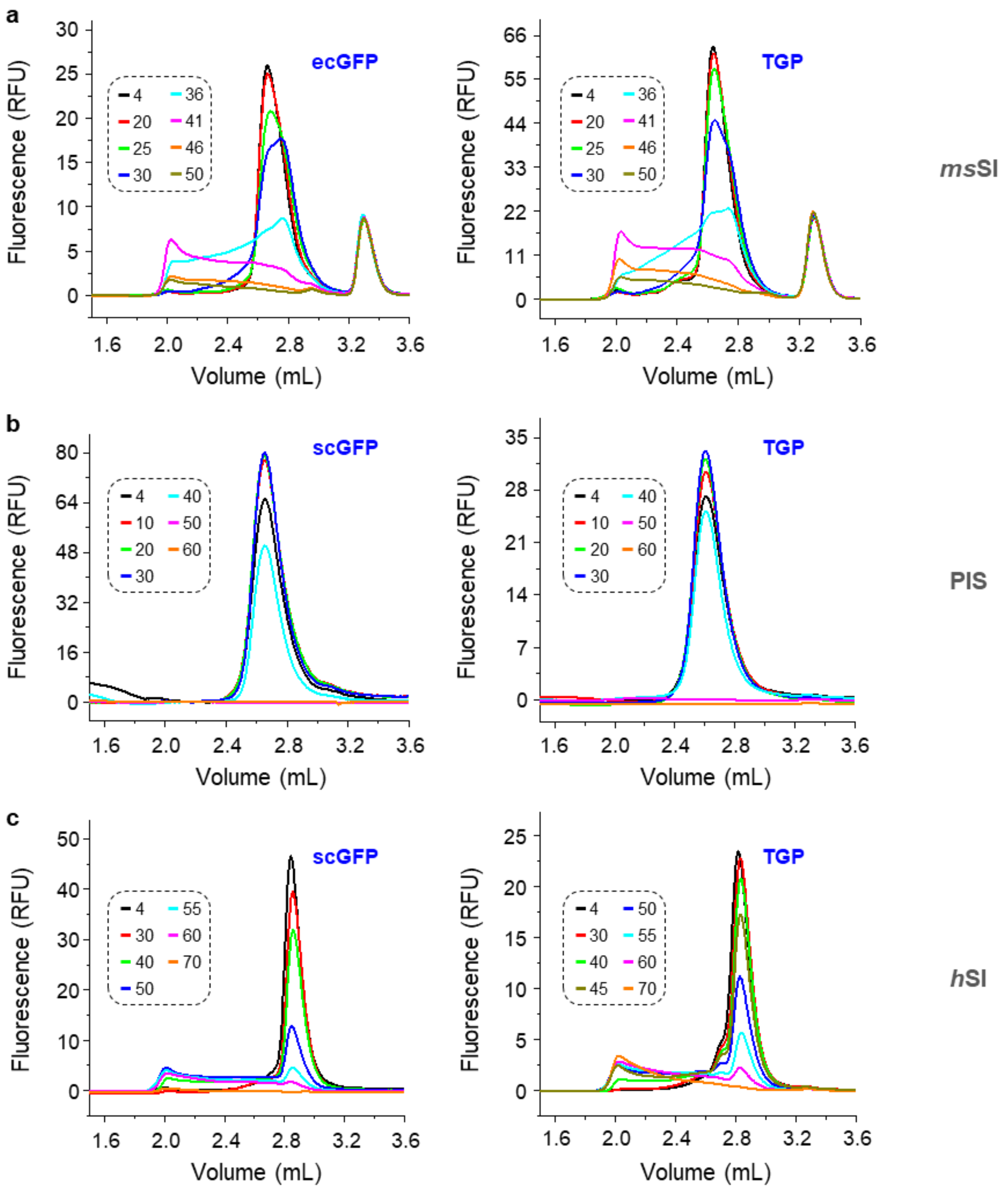

- continued on the next page

- continued from the previous page

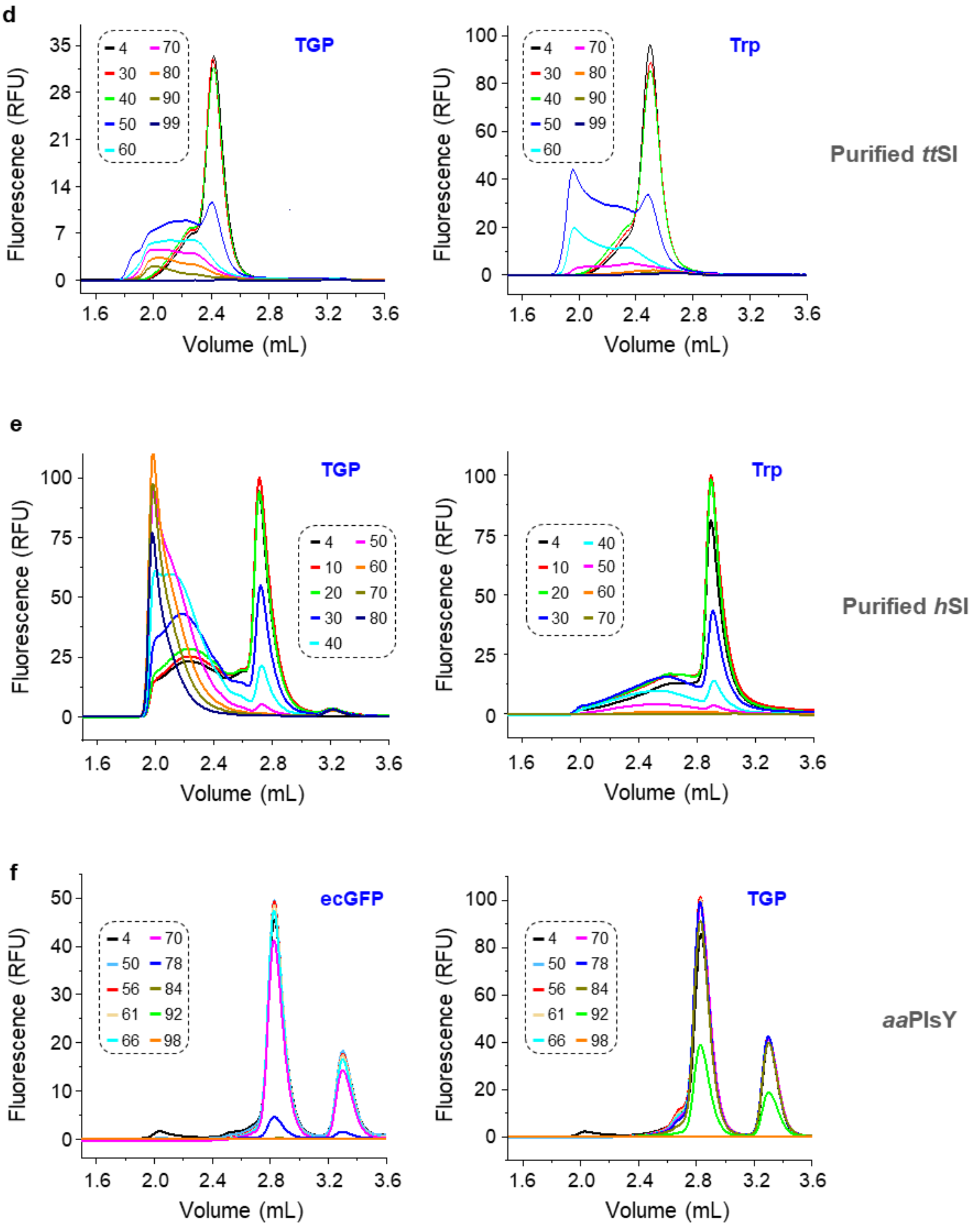

Supplementary Fig. 3. Original FSEC traces for FSEC-TS assays of membrane proteins. (**a**) *ms*SI. (**b**) PIS. (**c**) *h*SI. (**d**) *tt*SI. (**e**) *h*SI. (**f**) *aa*PlsY. For fluoreceent protein-based FSEC-assay, unpurified sample were used except for **d** and **e**. Color coding for heating temperatures within the same membrane protein is the same, as indicated in the dashed boxes. Abbreviations: FSEC, fluorescence-detection size exclusion chromatography; FSEC-TS, FSEC-based thermostability assay; GFP, green fluorescent protein; TGP, thermostable GFP.

Related to Fig. 2.

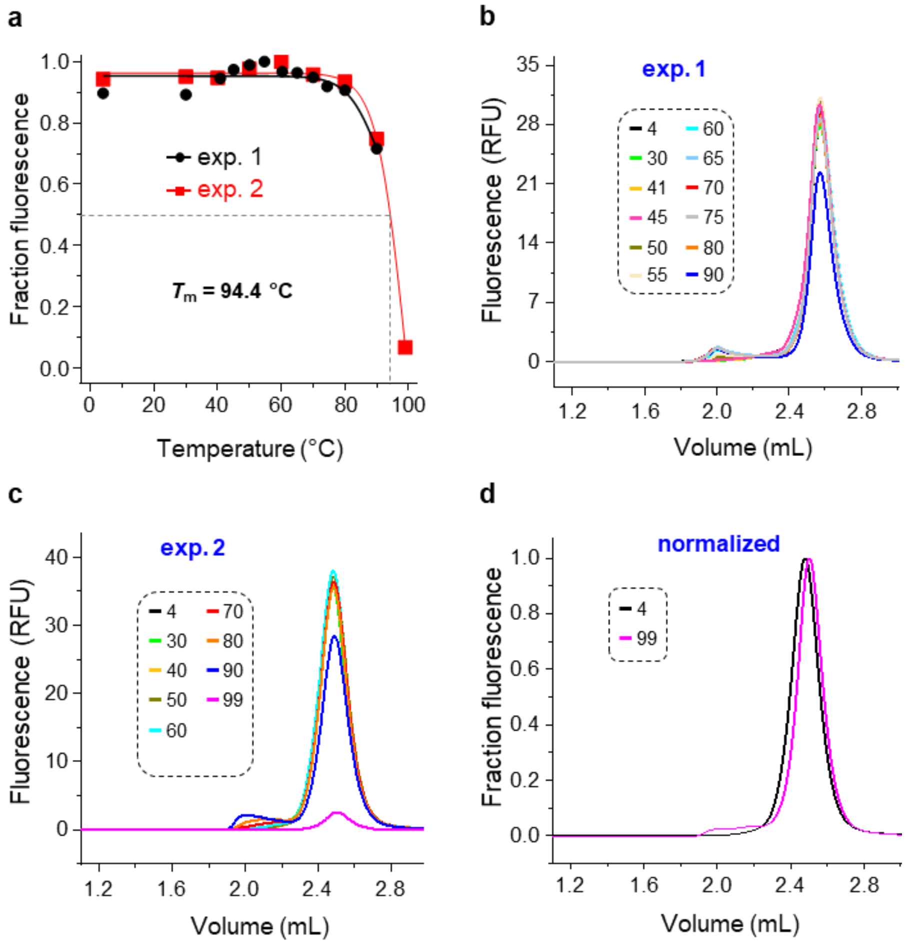

Supplementary Fig. 4. FSEC-TS assay of conSI. (**a**) The FSEC-TS curve of two independent experiments. (**b, c**) The original FSEC traces of conSI. Heating temperatures are indicated in each panel with the same color scheme (dashed boxes). conSI was heated in DDM detergent supplemented with 20 (w/w)% cholesteryl hemisuccinate (CHS). Consistent with literature that CHS generally enhances the stability of membrane proteins, the T_m obtained here was also 24 °C higher than what we previously obtained without CHS[1]. (**d**) Normalized FSEC profile of conSI treated at indicated temperatures. Abbreviations: FSEC, fluorescence-detection size exclusion chromatography; FSEC-TS, FSEC-based thermostability assay.

Related to Fig. 2.

```
                10         20         30         40         50         60
Sb44    QVQLVESGGG LVQAGGSLRL SCAASGFPVG RASMWWYRQA PGKEREWVAA ISSYGWVTAY
Sb66    QVQLVESGGG LVQAGGSLRL SCAASGFPVM ENSMYWYRQA PGKEREWVAA ITSQGSWTWY
Sb68    QVQLVESGGG LVQAGGSLRL SCAASGFPVN AVTMWWYRQA PGKEREWVAA IKSQGAGTWY
Sb92    QVQLVESGGG LVQAGGSLRL SCAASGFPVD TQWMHWYRQA PGKEREWVAA ISSTGRSTFY
        ********** ********** *********    :*:***** ********** *.* *  * *

                70         80         90        100        110
Sb44    ADSVKGRFTI SRDNAKNTVY LQMNSLKPED TAVYYCEVSV GTG-YRGQGTQ VTVS
Sb66    ADSVKGRFTI SRDNAKNTVY LQMNSLKPED TAVYYCHVSV GTN-YTGQGTQ VTVS
Sb68    ADSVKGRFTI SRDNAKNTVY LQMNSLKPED TAVYYCHVYV GAYRYEGRGTQ VTVS
Sb92    ADSVKGRFTI SRDNAKNTVY LQMNSLKPED TAVYYCTVYV GNR-YRGQGTQ VTVS
        ********** ********** ********** ******.* * *:  * *:*** ****
```

Orange: randomized residue without interaction.

Red: randomized residue with interaction.

Blue: framework residues with interaction.

Supplementary Fig. 5. Sequence alignment of TGP binders. Three CDRs are underlined. Asterisks indicate identical residues, while double and single dots refer to highly and modestly conserved residues, respectively. Color codes are explained in the figure. Abbreviations: TGP, thermostable green fluorescent protein.

Related to Fig. 8a.

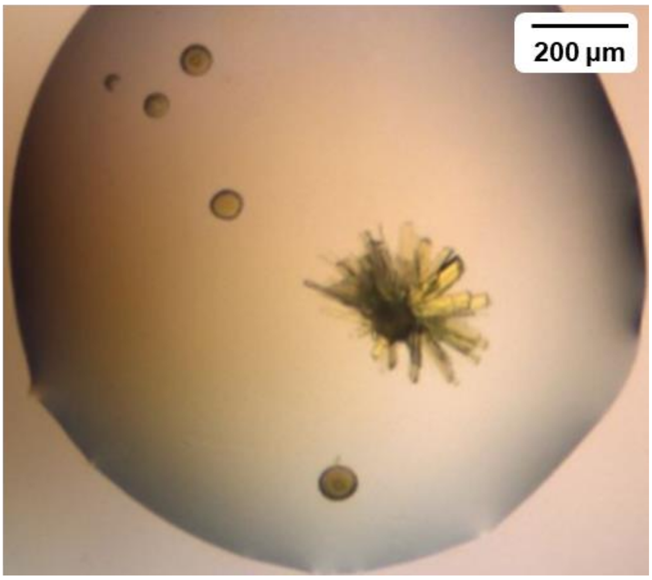

Supplementary Fig. 6. Crystal of TGP-Sb44. Crystals were grown in a sitting drop plate at 20 °C with a precipitant solution containing 0.2 M ammonium acetate, 25 %(w/v) polyethylene glycol 3,350, and 0.1 M Bis-Tris pH 6.5. Abbreviations: TGP, thermostable green fluorescent protein; sybody, synthetic nanobody.

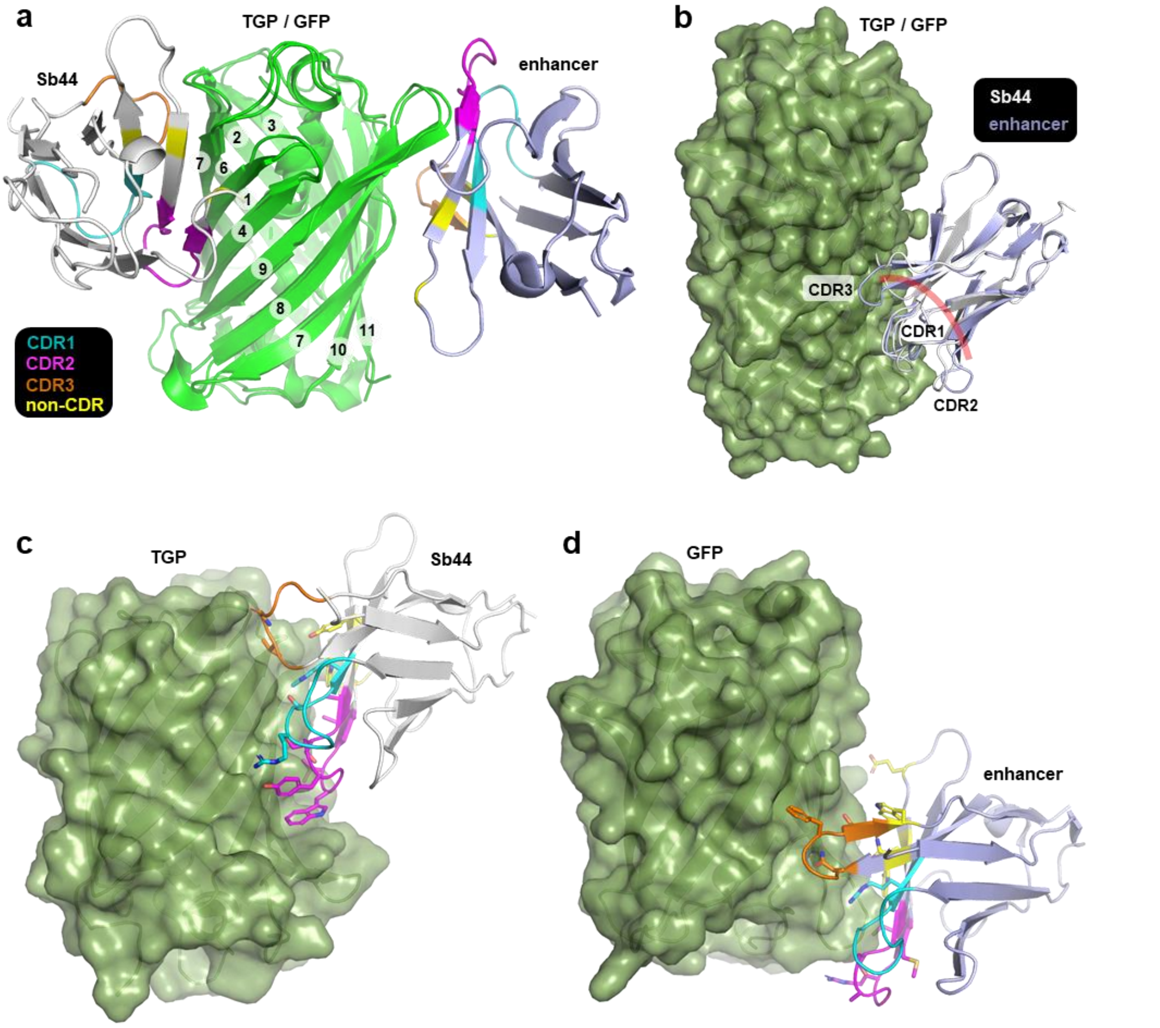

Supplementary Fig. 7. Differences and similarities between the binding modes of Sb44-TGP and enhancer-GFP. (**a**) Sb44 binds to TGP at the opposite side of the barrel compared with the enhancer to GFP. The structure of Sb44-TGP (6LZ2) was superposed with enhancer-GFP (3K1K)[2] via the two fluorecent proteins. Circled numbers indicate the 11 β-strands of both TGP and GFP. Non-CDR and CDRs are colored as indicated. The epitope for Sb44 consists of residues from β-strands 2, 4, 5, 6, and 9. The epitope for the enhancer consistes of residues from β-strands 7-10, with the majority from the loop between β8 and β9. (**b**) The similarities between Sb44 and the enhancer in binding to the fluorecent proteins. The structure of Sb44-TGP and enhancer-GFP were aligned based on the nanobody scaffold. Sb44 showed a similar concave paratope surface to the enhancer. In addition, non-CDR residues participated binding in both structures. (**c,d**) Separate views of Sb44-TGP and enhancer-GFP. Abbreviations: GFP, green fluorescent protein; TGP, thermostable GFP; sybody, synthetic nanobody; CDR, complementarity-determing region.

Related to Fig. 8c.

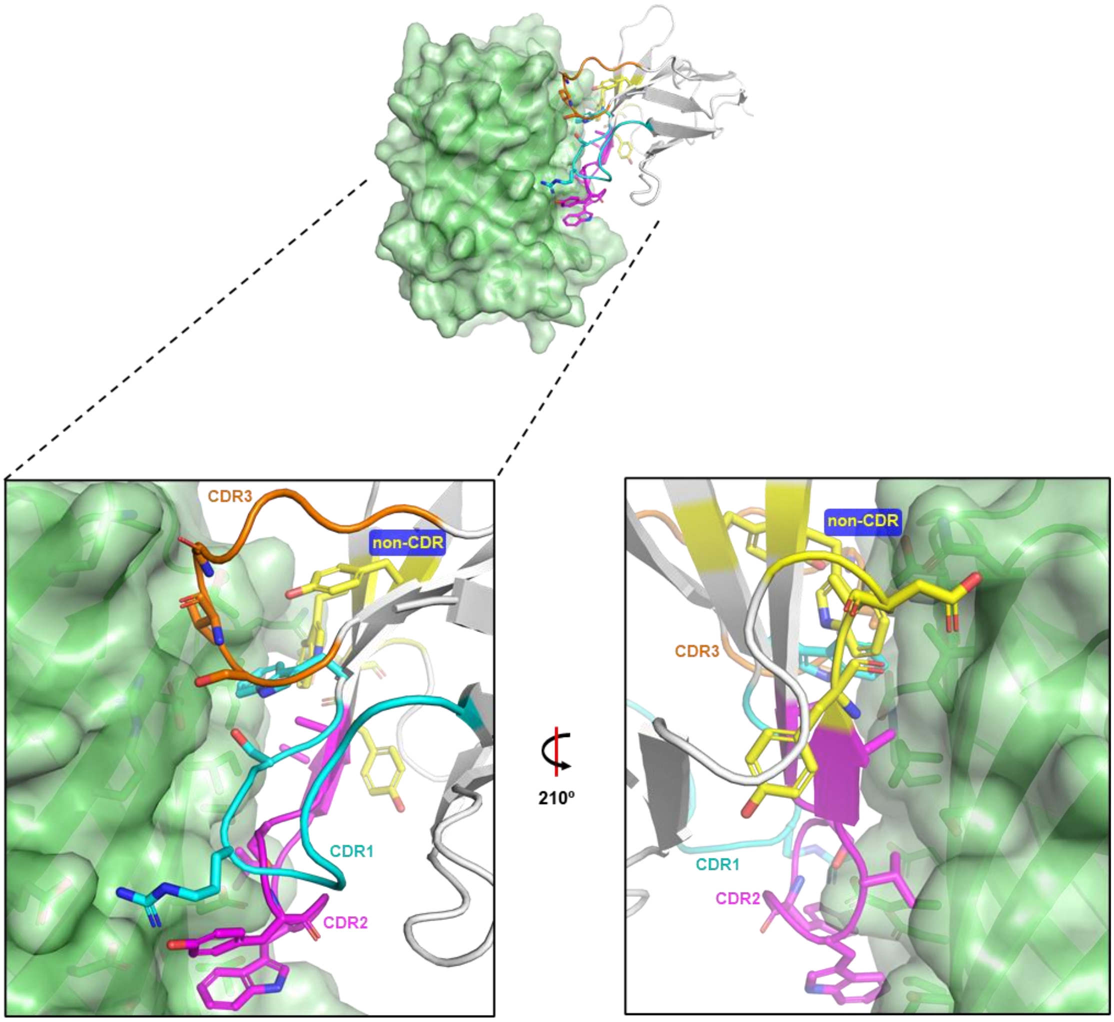

Supplementary Fig. 8. Overview and expanded view of interactions between Sb44 and TGP at two different angles. Abbreviations: TGP, thermostable green fluorescent protein; sybody, synthetic nanobody.

Related to Fig. 8c.

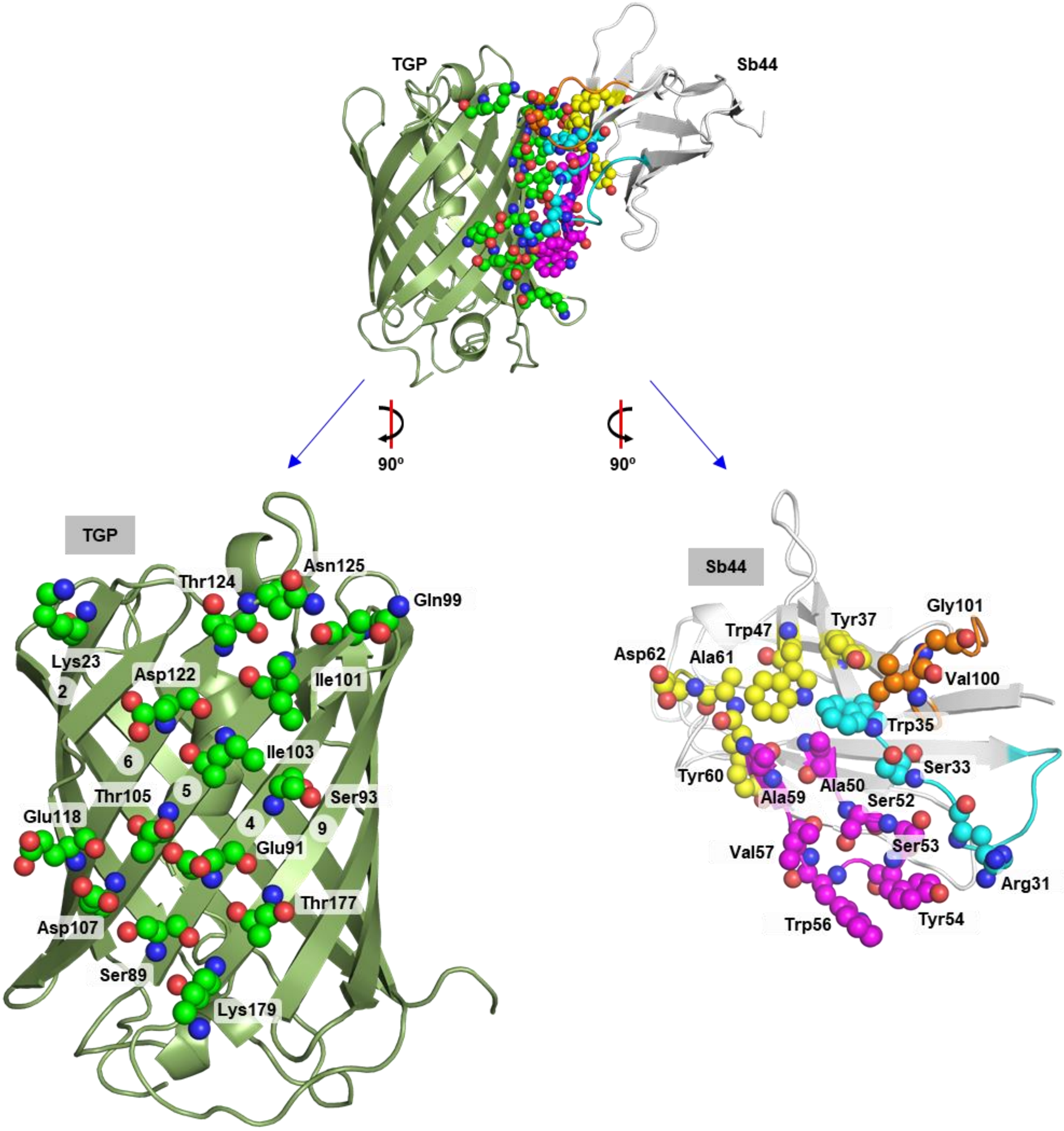

Supplementary Fig. 9. 'Open-book' representation of the interacting residues involved in Sb44-TGP binding. Circled numbers indicate TGP β-strands that contain interface residues. Abbreviations: TGP, thermostable green fluorescent protein; sybody, synthetic nanobody.

Related to Fig. 9c.

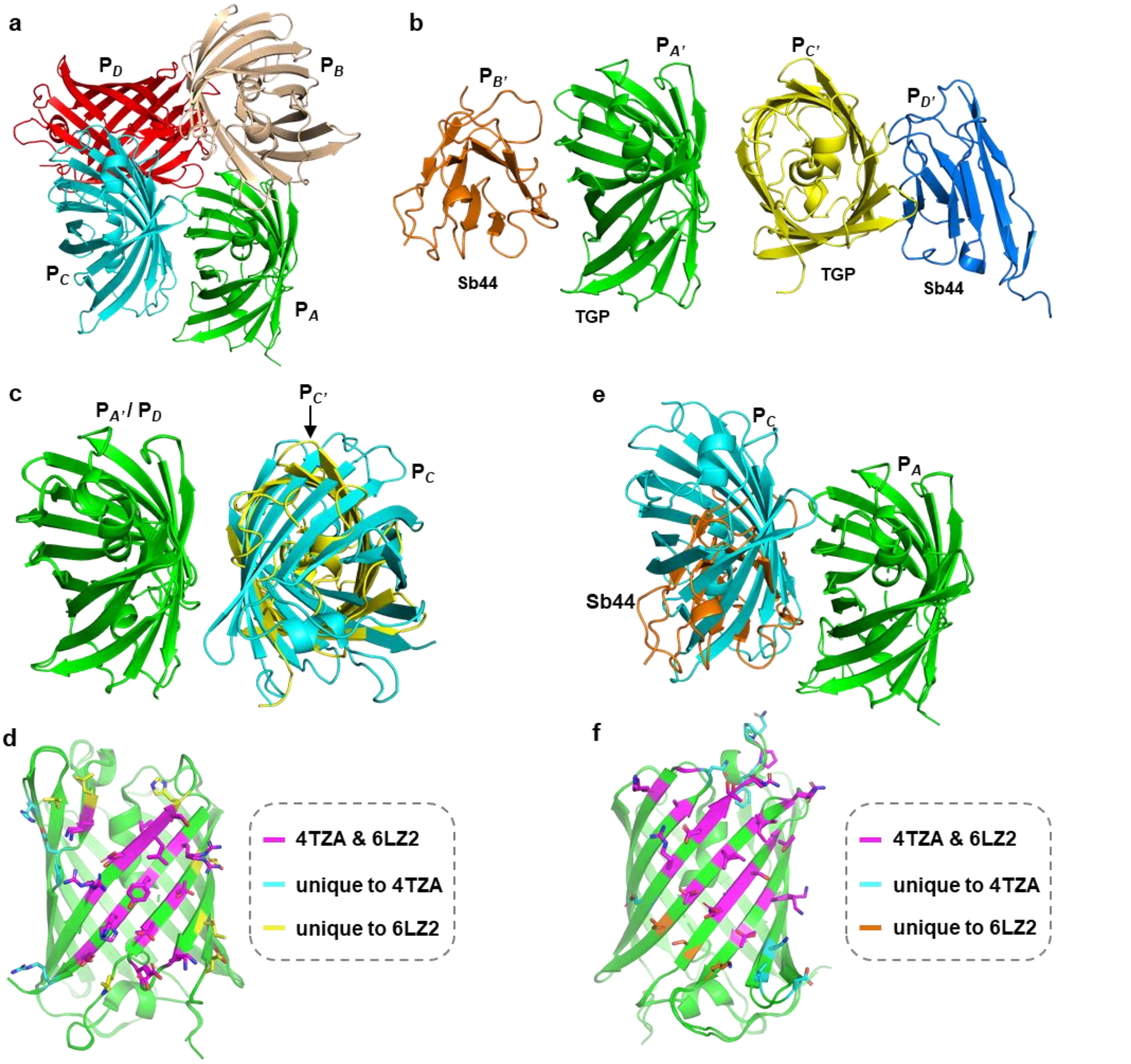

Supplementary Fig. 10. Overlap of interacting interfaces between the previous TGP structure and the current TGP-Sb44 structure. Overview of TGP alone[3] (PDB 4TZA) (**a**) and the TGP-Sb44 structure (PDB 6LZ2) (**b**) with labels for protomers (chains). The two TGP protomers in 6LZ2 assemble at the same site as observed in 4TZA but with a different orientation (**c**), showing an overall similar interacting surface (**d**). Sb44 binds to TGP in 6LZ2 at the same site as P_C binds to P_A in 4TZA (**e**) with an overlapping surface (**f**). Residues that are common or unique to each structure are color-coded as indicated in the box. Abbreviations: TGP, thermostable green fluorescent protein; sybody, synthetic nanobody.

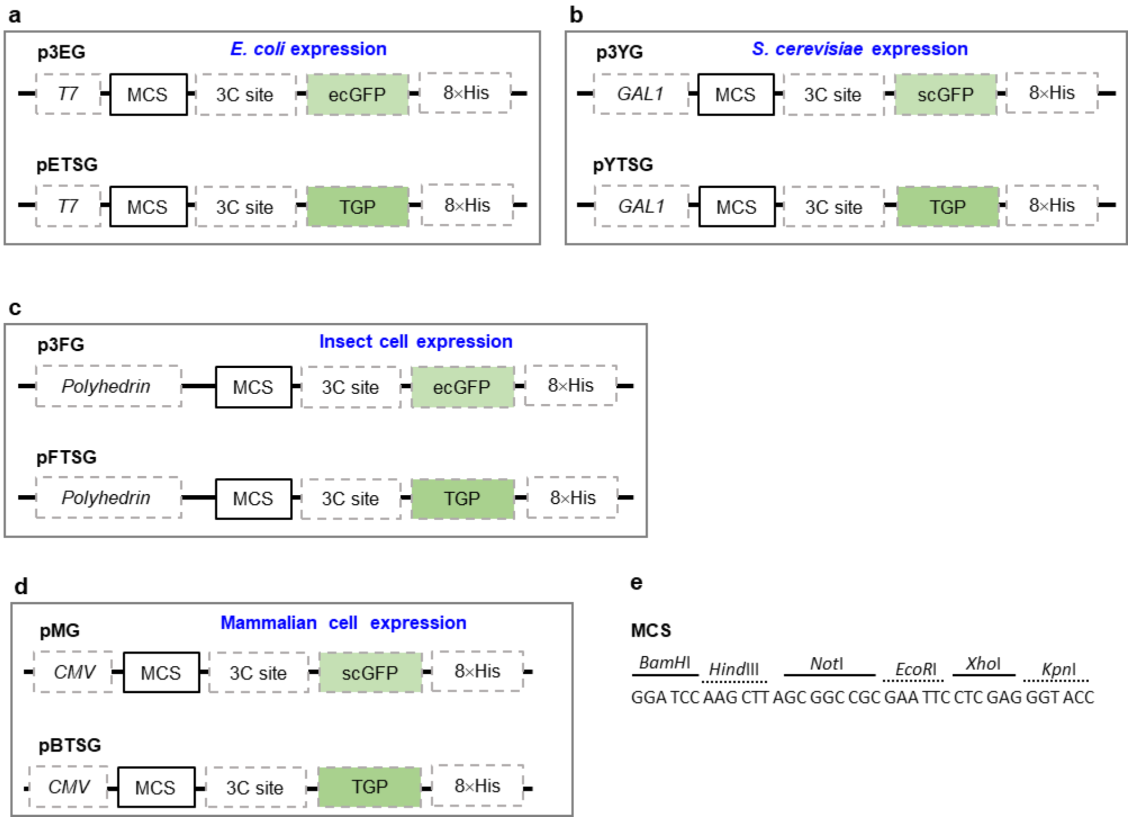

Supplementary Fig. 11. Vectors for expression of membrane proteins with a fluorescent protein tag. Simplified maps of the expression vectors for the *E. coli* (**a**), *S. cerevisiae* (**b**), insect cell (**c**), and mammalian system (**d**). Vector names are labeled at the left corner of each map. A cleavage site for the 3C protease was included for all vectors. Promoters were highlighted in italic fonts. A polyhistidine tag was included at the C-terminus of fluorescent proteins for purification with immobilized metal affinity chromatography. (**e**) The sequence of the uniformized multi-cloning site (MCS) with the restriction enzyme sites labeled above. Abbreviations: TGP, thermostable green fluorescent protein; GFP, green fluorescent protein.

Supplementary References

1. Yao, H., Cai, H. & Li, D. Thermostabilization of Membrane Proteins by Consensus Mutation: A Case Study for a Fungal Delta8-7 Sterol Isomerase. *J Mol Biol* **432**, 5162-5183 (2020).
2. Kirchhofer, A. et al. Modulation of protein properties in living cells using nanobodies. *Nat Struct Mol Biol* **17**, 133-138 (2010).
3. Close, D. W. et al. Thermal green protein, an extremely stable, nonaggregating fluorescent protein created by structure-guided surface engineering. *Proteins* **83**, 1225-1237 (2015).
