# Supplementary material for: An improved fluorescent tag and its nanobodies for membrane protein expression, stability assay, and purification
Source: Commun Biol. 2020 Dec 10;3:753. doi: 10.1038/s42003-020-01478-z (PMC7729955; doi:10.1038/s42003-020-01478-z)
Supplement: Supplementary file 2 — Description of Additional Supplementary Files [file 42003_2020_1478_MOESM2_ESM.pdf]

Description of Additional Supplementary Files

File Name: Supplementary Data 1
Description: Source data for Fig. 1a, 1c, 1d, 1e, Fig. 2, Fig. 3a, 3b, 3e-3h, Fig. 4a, 4b, 4e-4h, Fig. 5a, 5c, 5d, 5e, 5g, 5h, Fig. 6a, 6c, 6d, 6e, 6g, 6h, and Fig. 7c-7f in an Excel file.
