# Supplementary material for: An improved fluorescent tag and its nanobodies for membrane protein expression, stability assay, and purification
Source: Commun Biol. 2020 Dec 10;3:753. doi: 10.1038/s42003-020-01478-z (PMC7729955; doi:10.1038/s42003-020-01478-z)
Supplement: Supplementary file 5 — Reporting Summary [file 42003_2020_1478_MOESM5_ESM.pdf]

nature research

Corresponding author(s): DIANFAN LI

Last updated by author(s): Nov 3, 2020

Reporting Summary

Nature Research wishes to improve the reproducibility of the work that we publish. This form provides structure for consistency and transparency in reporting. For further information on Nature Research policies, see our Editorial Policies and the Editorial Policy Checklist.

Statistics

For all statistical analyses, confirm that the following items are present in the figure legend, table legend, main text, or Methods section.

n/a	Confirmed	
☐	☒	The exact sample size (n) for each experimental group/condition, given as a discrete number and unit of measurement
☐	☒	A statement on whether measurements were taken from distinct samples or whether the same sample was measured repeatedly
☒	☐	The statistical test(s) used AND whether they are one- or two-sided<br>*Only common tests should be described solely by name; describe more complex techniques in the Methods section.*
☒	☐	A description of all covariates tested
☒	☐	A description of any assumptions or corrections, such as tests of normality and adjustment for multiple comparisons
☐	☒	A full description of the statistical parameters including central tendency (e.g. means) or other basic estimates (e.g. regression coefficient) AND variation (e.g. standard deviation) or associated estimates of uncertainty (e.g. confidence intervals)
☒	☐	For null hypothesis testing, the test statistic (e.g. F, t, r) with confidence intervals, effect sizes, degrees of freedom and P value noted<br>*Give P values as exact values whenever suitable.*
☒	☐	For Bayesian analysis, information on the choice of priors and Markov chain Monte Carlo settings
☒	☐	For hierarchical and complex designs, identification of the appropriate level for tests and full reporting of outcomes
☒	☐	Estimates of effect sizes (e.g. Cohen's d, Pearson's r), indicating how they were calculated

Our web collection on statistics for biologists contains articles on many of the points above.

Software and code

Policy information about availability of computer code

Data collection	Fluorescence counting, Softmax Pro 7.0; Fluorescence-detection size exclusion chromatography, Lab Solutions 5.87; Preparative size exclusion chromatography, ChromLab 3.3.0.09; In-gel fluorescence, Image Lab 5.2; Biolayer inteferometry assay, Octet Data Analysis 10.0; Crystal imaging, RockImager 3.4.3.1; X-ray diffraction, Blue Ice.
Data analysis	Curve fitting for melting temperature, OriginPro 9.6.0.172; In-gel fluorescence intensity analysis, Image Lab 5.2; Biolayer inteferometry analysis, Octet Data Analysis 10.0; X-ray diffraction data processing, XDS; X-ray diffraction data scaling and molecular replacement, Aimless/ Phaser in ccp4 7.0.078; 3D-model building, Coot 0.8.9.2; Structure refinement, Phenix 1.9-1692; Structure visualization, PyMOL 2.3.3.

For manuscripts utilizing custom algorithms or software that are central to the research but not yet described in published literature, software must be made available to editors and reviewers. We strongly encourage code deposition in a community repository (e.g. GitHub). See the Nature Research guidelines for submitting code & software for further information.

Data

Policy information about availability of data

All manuscripts must include a data availability statement. This statement should provide the following information, where applicable:

- Accession codes, unique identifiers, or web links for publicly available datasets
- A list of figures that have associated raw data
- A description of any restrictions on data availability

Atomic coordinates and structure factors for the reported TGP-Sb44 structure are deposited in the Protein Data Bank (PDB) under accession codes of 6LZ2. Relevant plasmids and sequences have been deposited in Addgene (www.addgene.org) with the following IDs: pETSG, 159418; pYTSG, 159419; pFTSG, 162389; pBTSG, 159420; pSB_init_Sb44, 159421; pSB_init_Sb66, 159422; pSB_init_Sb68, 159423; pSB_init_Sb92, 159424. The raw data for Fig. 1a, 1c, 1d, 1e, Fig. 2, Fig. 3a, 3b, 3e-3h, Fig. 4a, 4b, 4e-4h, Fig. 5a, 5c, 5d, 5e, 5g, 5h, Fig. 6a, 6c, 6d, 6e, 6g, 6h, and Fig. 7c-7f are available through Supplementary Data. There is no restrictions on

data availability.

Field-specific reporting

Please select the one below that is the best fit for your research. If you are not sure, read the appropriate sections before making your selection.

☒ Life sciences ☐ Behavioural & social sciences ☐ Ecological, evolutionary & environmental sciences

For a reference copy of the document with all sections, see nature.com/documents/nr-reporting-summary-flat.pdf

Life sciences study design

All studies must disclose on these points even when the disclosure is negative.

Sample size	Not applicable
Data exclusions	No data were excluded from the analysis
Replication	Most multi-point melting-curve experiments are performed once except for Fig. 2e where three replicates were performed to confirm the rise of fluorescent signal before falling. Data for expression level are from three independent experiments. Details are included in the Method, figure legends, and table footnotes.
Randomization	Not applicable
Blinding	Not applicable

Reporting for specific materials, systems and methods

We require information from authors about some types of materials, experimental systems and methods used in many studies. Here, indicate whether each material, system or method listed is relevant to your study. If you are not sure if a list item applies to your research, read the appropriate section before selecting a response.

Materials & experimental systems

n/a	Involved in the study	
☐	☒	Antibodies
☐	☒	Eukaryotic cell lines
☒	☐	Palaeontology and archaeology
☒	☐	Animals and other organisms
☒	☐	Human research participants
☒	☐	Clinical data
☒	☐	Dual use research of concern

Methods

n/a	Involved in the study	
☒	☐	ChIP-seq
☒	☐	Flow cytometry
☒	☐	MRI-based neuroimaging

Antibodies

Antibodies used	Nanobodies against the thermostable green fluorescence protein (TGP) were selected from a synthetic library. Sequence information are provided in the manuscript. Plasmids are deposited in Addgene.
Validation	The binding between the nanobodies and TGP were validated by gel-filtration assays, biolayer inteferometry binding assays, and structural studies.

Eukaryotic cell lines

Policy information about cell lines

Cell line source(s)	Saccharomyces cerevisiae BCY123 (MATα pep4::HIS3 prb::LEU2 bar1:HISG lys2::GAL1/10-GAL4 can1 ade2 ura3 leu2-3 112 trp1), Prof. Jinqiu Zhou's lab at the authors' institute; Pichia pastoris GS115 (his4), lab collection; Insect sf9 cells, lab collection; Expi293F, Thermo Fisher Scientific.
Authentication	The yeast cells have been tested for their auxotrophic phenotype in our lab. We did not test the authenticity of the Expi293F cells.
Mycoplasma contamination	Cell lines were not tested for mycoplasma contamination.
Commonly misidentified lines (See ICLAC register)	Not applicable
